# A note of caution on the diagnosis of Martin-Probst syndrome by the detection of the p.D59G mutation in the *RAB40AL* gene

**DOI:** 10.1007/s00431-014-2452-x

**Published:** 2014-11-05

**Authors:** Monika Ołdak, Ewelina Ruszkowska, Agnieszka Pollak, Agnieszka Sobczyk-Kopcioł, Cezary Kowalewski, Aleksandra Piwońska, Wojciech Drygas, Rafał Płoski

**Affiliations:** 1 Department of Genetics, Institute of Physiology and Pathology of Hearing, Mokra 17, Kajetany, 05-830 Nadarzyn, Poland; 2 Department of Histology and Embryology, Medical University of Warsaw, Warsaw, Poland; 3 Department of General Biology and Parasitology, Medical University of Warsaw, Warsaw, Poland; 4 Department of Dermatology and Immunodermatology, Medical University of Warsaw, Warsaw, Poland; 5 Department of Epidemiology, Cardiovascular Diseases Prevention and Promotion of Health, Institute of Cardiology, Warsaw, Poland; 6 Department of Medical Genetics, Medical University of Warsaw, Pawińskiego 3c, 02-016 Warsaw, Poland

**Keywords:** *RAB40AL*, p.D59G, Mutation, Martin-Probst syndrome, Whole exome sequencing

## Abstract

Martin-Probst syndrome (MPS) is an X-linked multisystem neurodevelopmental disorder, reported to be caused by the p.D59G mutation in *RAB40AL*. Whereas evidence against the pathogenic role of p.D59G has been published, the presence of *RAB40AL* p.D59G continues to be used as a support for MPS diagnosis. Our purpose was to provide further arguments for excluding pathogenicity of *RAB40AL* p.D59G. We detected p.D59G in two healthy males ascertained as family members of p.D59G carriers who underwent whole exome sequencing for diagnostic reasons. Furthermore, we found that p.D59G was present in 2.86 % (4/140) of randomly selected Polish males with higher education.

*Conclusion*: Our findings are inconsistent with a causative effect of *RAB40AL* p.D59G on cognitive impairment combined with severe to profound bilateral hearing loss but indicate that p.D59G is a common genetic variation. Our data emphasize the need for genotyping large sample sizes of diverse populations as a basic tool in determining variant pathogenicity.

## Introduction

Martin-Probst syndrome (MPS, MIM %300519) is an X-linked neurodevelopmental disorder characterized by sensorineural hearing loss, intellectual disability and a variety of dysmorphic features [5]. MPS has been originally described in three males from a single family. Whereas evidence against the pathogenic role of p.D59G has been published [2, 6], the presence of *RAB40AL* (MIM *300405) p.D59G continues to be used to support MPS diagnosis as evidenced by a recent publication by Lee et al. describing the fourth male with MPS [4]. Since the overlap of symptoms between the case reported by Lee et al. [4] and originally described patients [5] was only partial (out of nine features present in all three affected subjects from the first family [5], only four were present in the patient of Lee et al. [4]), the diagnosis rested mainly on the detection of the p.D59G mutation in the *RAB40AL* gene, which has been proposed to cause MPS [1].

The *RAB40AL* gene (NG_017150.1) contains one exon of 1713-bp length and more than 70 different single nucleotide polymorphisms (SNP) in the coding sequence. Many of them occur at a low frequency and are predicted to be deleterious by in silico analysis (www.ensembl.org, accessed 10/2014). However, none of them has been associated with any disease. The only mutation identified in *RAB40AL* is c.176_177delACinsGA found to segregate with MPS. The variant represents a dinucleotide change and contains a NM_001031834.1:c.176A>G transition (rs145606134) co-occurring with a NM_001031834.1:c.177C>A transversion (rs138133927). These substitutions, when present together, are predicted to cause a replacement of aspartic acid by glycine (GAC>GGA) at codon 59 (p.D59G) [1].

Whereas the report by Lee et al. [4] is potentially important for refining the spectrum of MPS symptoms, our study presents new data casting doubt on the pathogenicity of *RAB40AL* p.D59G in MPS.

## Materials and methods

### Patients

**Fig. 1:**
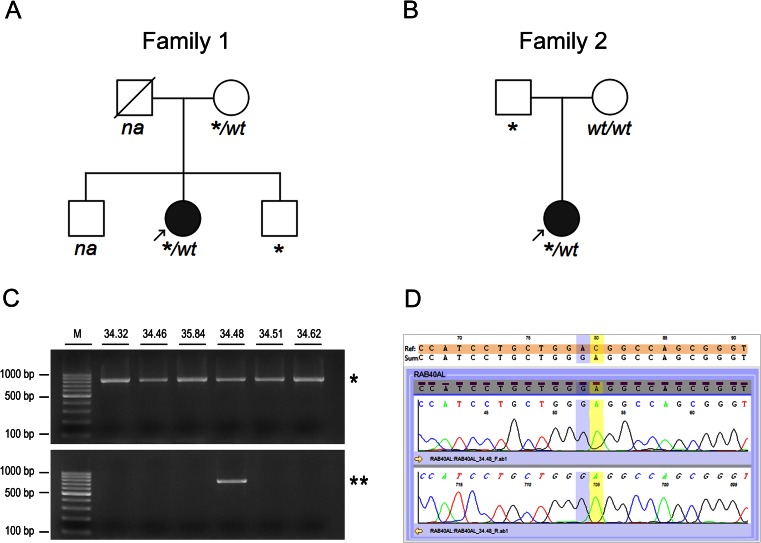
Detection of *RAB40AL* p.D59G in the studied individuals. (**a**, **b**) Pedigree of the two Polish families investigated in the study showing the index patient (*arrow*) and segregation of p.D59G (*) within the families; *wt* indicates the presence of the *RAB40AL* wild-type allele, *na* indicates family members not available for genetic examination. *Filled symbols* denote individuals affected with the (**a**) Pendred syndrome or (**b**) congenital ichthyosis, *open symbols* denote unaffected individuals. (**c**) *RAB40AL* was PCR amplified in all tested individuals (812-bp product (*)), specific amplification of the *RAB40AL* c.176_177delACinsGA variant was identified in some of them (751-bp product (**), *lane 5*). One representative gel is shown. *M* indicates the DNA molecular weight marker (*lane 1*). (**d**) Direct DNA sequencing of the *RAB40AL* gene confirmed the presence of c.176A>G transition (rs145606134, *shaded blue*) and c.177C>A transversion (rs138133927, *shaded yellow*) predicting the amino acid change p.D59G (GAC>GGA). One representative electropherogram is shown

Genomic DNA was isolated from peripheral blood from all available family members (*n* = 6) from two unrelated Polish families. The index patient in the first family was a 36-year-old female with profound bilateral sensorineural hearing loss from infancy and hypothyroid goiter treated with thyroxin from the age of 15 years. Whole exome sequencing (WES) in the patient identified two heterozygous mutations in the *SLC26A4* gene (MIM *605646), which allowed the diagnosis of Pendred syndrome (PDS #274600) (Fig. 1a). The second index patient was a female with congenital ichthyosis (MIM #242100) most probably resulting from recessive mutations in the *ALOX12B* gene (MIM *603741) (Fig. 1b). Both index patients were otherwise healthy and their family history was unremarkable. Males in both families did not present with any symptoms or signs suggestive of MPS. The brother of the first index patient graduated from a secondary technical school. The father of the second index patient used to be a competitive swimmer and currently working as a lifeguard.

A set of DNA samples (*n* = 140) from males with a completed higher education was selected from a repository of DNA samples obtained from randomly chosen permanent Polish residents and used for studying genetic risk factors for cardiovascular diseases [3]. All examined individuals gave a written informed consent for genetic testing and the study was approved by the local ethics committees.

### Methods

Genotyping of the dinucleotide substitution (c.176_177delACinsGA) in the *RAB40AL* leading to p.D59G was performed as described previously using allele-specific PCR followed by direct Sanger sequencing [6].

## Results

The p.D59G in the *RAB40AL* gene was identified by WES sequencing in two female probands with distinct medical problems. Further analysis revealed that in the first family, p.D59G was inherited by the index patient and her brother from their healthy mother (Fig. 1a) whereas in the second family, the variant was transmitted to the index patient from her healthy father (Fig. 1b). Noteworthy, both males hemizygous for the p.D59G variant in these families do not have hearing loss, intellectual disability or other phenotypic feature reported in patients with MPS and they consider themselves generally healthy.

Studying the prevalence of the p.D59G in a cohort of Polish males with a completed higher education, we identified the *RAB40AL* variant in 4 out of 140 individuals. It corresponds to an allele frequency of 2.86 % in males, which is typical for a common genetic variation (Fig. 1c, d).

## Discussion

Our data provide further strong evidence against the pathogenicity of the *RAB40AL* p.D59G variant in MPS. Performing WES, we have detected the *RAB40AL* p.D59G variant in two unrelated female patients with a clinical diagnosis other than MPS. As MPS is an X-linked recessive disorder, we have studied the segregation of p.D59G in these families and found that none of the male family members hemizygous for p.D59G had any medical problems, and there was no evidence of MPS in these individuals. Considering that intellectual disability is one of the main clinical features of MPS, we aimed to examine the prevalence of the *RAB40AL* genetic variant in a cohort of Polish males with a completed higher education. The resulting data showed a high prevalence (2.86 %) of p.D59G in this group that is typical of a benign polymorphism.

Based on recently proposed guidelines for the assessment of genetic variants potentially involved in X-linked intellectual disability, detection of a variant in more than one male or individual of the general population argues against its pathogenic role [7]. In the ESP6500 database (NHLBI GO Exome Sequencing Project http://evs.gs.washington.edu/EVS/), a large exome database, the c.176A>G substitution (rs145606134) was detected in 2 and the c.177C>A substitution (rs138133927) in 1 out of 6728 alleles of European Americans (accessed 10/2014).

Furthermore, the prevalence of p.D59G is apparently quite high in German and Polish populations. Kalscheuer et al. identified the p.D59G variant in 4 out of 446 index patients with intellectual disability, although in all cases its causative role was unlikely [2]. Our group recently found p.D59G by WES in two males in whom the diagnosis of MPS was excluded. Furthermore, by screening control DNA samples (*n* = 810) from a general Polish population, we found p.D59G in 8/405 males and 12/405 females, indicating that it has a high prevalence (2.47 %) typical of a benign polymorphism [6].

These data suggest that the presence of *RAB40AL* p.D59G in the patient described by Lee et al. [4] cannot be regarded as a proof for the diagnosis of MPS. Thus, before the true genetic determinant of MPS is found and appropriately confirmed, caution should be exerted in accepting that the patient described by Lee et al. [4] has MPS as a consequence of *RAB40AL* p.D59G.

In the context of previous work [1], our study emphasizes the importance of investigating large sample sizes of diverse populations as a basic tool in determining variant pathogenicity. Considering the high prevalence of *RAB40AL* p.D59G in the general population and an increasing use of whole exome sequencing in the clinic, our results should help to avoid diagnostic pitfalls.
